# Body Mass Index of 92,027 patients acutely admitted to general hospitals in Denmark: Associated clinical characteristics and 30-day mortality

**DOI:** 10.1371/journal.pone.0195853

**Published:** 2018-04-16

**Authors:** Sigrid Bjerge Gribsholt, Lars Pedersen, Bjørn Richelsen, Olaf Dekkers, Reimar Wernich Thomsen

**Affiliations:** 1 Department of Clinical Epidemiology, Aarhus University Hospital, Aarhus, Denmark; 2 Department of Endocrinology and Internal Medicine, Aarhus University Hospital, Aarhus, Denmark; 3 Department of Clinical Epidemiology and Internal Medicine, Leiden University Medical Centre, Leiden, The Netherlands; Dasman Diabetes Institute, KUWAIT

## Abstract

**Background:**

Data are sparse on the range of BMI among patients acutely admitted to general hospitals. We investigated BMI values and associated patient characteristics, reasons for hospital admission, and mortality in Denmark.

**Methods:**

We identified all persons with an acute inpatient admission 2011–2014 in Central Denmark Region and assessed BMI measurements recorded in the Clinical Information System. We used cross-sectional and cohort analyses to examine the BMI distribution and its association with demographic characteristics, comorbidities, medication use, tobacco smoking, reasons for admission, and 30-day mortality.

**Results:**

Among 92,027 acutely admitted patients (median age 62 years, 49% female) with a BMI measurement, 4% had a BMI (kg/m^2^) <18.5, 42% a BMI between 18.5 and 25, 34% a BMI between 25 and 30, and 20% a BMI ≥30. Compared with normal-weight patients, 30-day mortality was high among patients with BMI <18.5 (7.5% vs. 2.8%, age- and smoking-adjusted odds ratio (aOR) 2.4; 95% confidence interval (CI): 2.0–2.9, whereas patients with overweight (aOR 0.7; 95% CI: 0.6–0.8) and obesity class I (aOR 0.8; 95% CI: 0.6–0.9)). Compared with the total population, patients with BMI <18.5 were older (68 years median); more were female (73%); more had comorbidities (Charlson Comorbidity Index score >0 in 42% vs. 33% overall), more were current smokers (45% vs. 27% overall), and acute admissions due to respiratory diseases or femoral fractures were frequent. In contrast, patients with BMI ≥30 were relatively young (59 years median), fewer smoked (24%): type 2 diabetes, sleep disorders, cholelithiasis, and heart failure were frequent diagnoses. Prevalence of therapies for metabolic syndrome, pain, and psychiatric disorders increased with higher BMI, while patients with BMI <18.5 frequently used asthma medications, glucocorticoids, and antibiotics.

**Conclusion:**

In patients acutely admitted to general hospitals, reasons for hospital admission and associated clinical characteristics differ substantially according to BMI range. BMI <18.5 is a clinical predictor of high short-term mortality.

## Introduction

Both a low Body Mass Index (BMI) (<18.5 kg/m^2^, underweight) and a high BMI (≥30 kg/m^2^, obesity) are risk factors for increased morbidity, mortality, and adverse outcome of many diseases in the general population.[1–5] Despite that, knowledge is sparse on BMI values in patients acutely admitted to general hospitals, and on clinical characteristics and outcomes associated with different BMI levels. Earlier studies have suggested a protective effect of overweight or moderate obesity in patients acutely admitted to hospital with heart failure, hip fracture, or sepsis–the so-called obesity paradox.[6–11] In general population studies, newer meta-analyses have challenged the presence of any obesity paradox[5,12] suggesting that previous findings have been biased by differences in clinical and lifestyle characteristics associated with different BMI ranges.[13] In general patient populations, such data are scarce.

Large-scale electronic patient data from clinical information systems constitute a potentially valuable and cost-efficient method of collecting data on patients’ BMI, both for health care quality monitoring and for prognostic epidemiological research.[14] Studies have discussed the potential of using electronic health records, finding great opportunities of investigating disease development and treatment using electronic health records.[15–17] The Central Denmark Region Clinical Information System (CDRCIS) contains readily available data on BMI covering the entire hospital population in the Region, with virtually complete patient follow-up.[18]

In the present study, we aimed to examine BMI among patients acutely admitted to general hospitals and the association of BMI with 30-day mortality. We also examined characteristics associated with different BMI levels, including demographic characteristics, comorbidities, medication use, tobacco smoking, and reasons for admission.

## Methods

The Central Denmark Region has a mixed rural and urban population of approximately 1.27 million persons. In Denmark, medical records of individual patients are tracked using civil personal registration numbers. These unique identifiers, encoding sex and date of birth, have been assigned to all Danish residents since 1968. In the present study, we used civil personal registration numbers to link data from the Danish National Patient Registry (DNPR),[14] the Civil Registration System (CRS),[18] the CDRCIS, and the Aarhus University Prescription Database (AUPD).[19]

### Study population

We used the DNPR to identify all individuals aged > = 18 years in the Central Denmark Region with one or more acute inpatient hospital admissions recorded between January 1, 2011 and December 31, 2014 (N = 242,637). We then identified all patients with a body mass index (BMI) measurement in the CDRCIS recorded during their first acute hospital admission in the study period (N = 92,027, 38%). We grouped these BMI measurement according to standard categories: underweight (<18.5 kg/m^2^), normal weight (18.5 to <25 kg/m^2^), overweight (25 to <30 kg/m^2^), obesity class I (30 to <35 kg/m^2^), obesity class II (35 to <40 kg/m^2^), and obesity class III (≥40 kg/m^2^).

### 30-day mortality

We ascertained complete 30-day mortality data from the Danish CRS system.[18]

### Reason for hospital admission

In each BMI category, we used the primary (first-listed) discharge diagnosis code recorded in the DNPR to identify the main reason for the first acute inpatient admission during the study period. We then examined 10 primary *International Classification of Diseases*, *Tenth Revision* (ICD-10) diagnoses that have been commonly related to either high or low BMI in the literature: femur fracture[20,21] abdominal pain, angina,[22] atrial fibrillation,[23] pneumonia,[24,25] chronic obstructive pulmonary disease [COPD], [5,26,27] type 2 diabetes [T2D],[28] erysipelas,[29,30] cholelithiasis,[31] and sleep disturbances [32] (see S1 Table for codes). Second, we grouped patients according to major disease categories in ICD-10 (S1 Table).

### Comorbidity

Based on DNPR data, we summarized each patient’s comorbidity history based on hospital contacts during the five years prior to the admission date, according to 19 disease categories included in the Charlson Comorbidity Index (CCI).[33] We grouped patients by overall level of comorbidity, defined as a CCI score of 0 (no comorbidity recorded), 1–2 (moderate comorbidity), and 3+ (severe comorbidity) (see S3 and S4 Tables).

### Smoking

We used data from the CDRCIS to categorize patients’ tobacco smoking status on the hospital admission date as follows: no smoking, current smoking, former smoking, or occasional smoking.

### Medication use

We obtained individual-level information from the AUPD on prescription medications redeemed within 180 days before the hospital admission, and classified patients as users/non-users of anti-hypertensive medications, glucose-lowering medications, lipid-lowering medications, antidepressant or anxiolytic medications, prescription painkillers, inhalants for obstructive airway diseases, glucocorticoids, and medications for gastric disorders (see S5 Table for codes).

### Statistical analyses

We first ascertained the BMI distribution in the study population. We then tabulated demographic variables (sex and age), CCI score (none, moderate, or severe), number of admissions, hospital and department type, and smoking status (categorized as above). We calculated 30-day mortality proportions for each predefined BMI category and estimated mortality odds ratios (ORs), using BMI 18.5 to <25 kg/m^2^ as reference category. We also calculated the OR adjusted for age and smoking status (adjusted OR, aOR), to assess if associations with BMI were independent of age and smoking. We refrained from further adjustments, as the order of factors in the causal pathway is impossible to disentangle in a cross-sectional design; factors such as comorbidities and medications may thus be mediators of any BMI effect on mortality rather than confounders. We presented graphically the proportions of users of selected medications and important causes of admission by BMI category, and further tabulated hospital contacts according to primary ICD-10 diagnosis chapters. We also calculated the prevalence ratios of causes of admission, using BMI 18.5 to <25 kg/m^2^ as reference category.

Our study followed the RECORD guidelines,[34] and all data were fully anonymized before we accessed them. The study was approved by the Danish Data Protection Agency (Record Number KEA-2016-26). According to Danish legislation, registry-based studies do not require separate approval from the Danish Scientific Ethics Committee, nor do they require a written consent from the patients. The source data files were kept by the Central Denmark region and only files with the needed variables were exported to the Department of Clinical Epidemiology for research purposes. Only structured data were exported and the unique personal identifier (the CPR number) were encrypted in all data files. Personal variables like names, addresses etc. were excluded in all data files before transmission. Patients did not provide a written consent as the The Danish Act on Processing of Personal Data (Persondataloven) provides the legal basis for the ability of public institutions, including universities, to retain person-identifiable health data for research purposes. In addition, use of these data required a project-specific permission from the Data Protection Agency (Datatilsynet, www.datatilsynet.dk).

## Results

### BMI distribution in hospitalized patients

Among 92,027 acutely admitted patients (median age 62 years, 50.5% male), the BMI distribution was as follows: underweight: 4.0%, normal weight: 41.8%, overweight: 33.8%, obesity class I: 13.9%, obesity class II: 4.4%, and obesity class III: 2.1% (Table 1).

**Table 1:** Characteristics of 92,027 patients acutely admitted to hospital in the Central Denmark Region between 2011 and 2014.

	Total	BMI<18.5 kg/m^2^	BMI 18.5 to 25 kg/m^2^	BMI 25 to 30 kg/m^2^	BMI 30 to 35 kg/m^2^	BMI 35 to 40 kg/m^2^	BMI > 40 kg/m^2^	Obesity, all, BMI > 30 kg/m^2^
**Overall:**	92,027 (100)	3701 (100)	38,446 (100)	31,093 (100)	12,810 (100)	4048 (100)	1929 (100)	18,787 (100)
**Sex**								
Female, n (%)	45,570 (49.5)	2709 (73.2)	21,001 (54.6)	12,682 (40.8)	5791 (45.2)	2213 (54.7)	1174 (60.9)	9178 (48.9)
Male, n (%)	46,457 (50.5)	992 (26.8)	17,445 (45.4)	18,411 (59.2)	7019 (54.8)	1835 (45.3)	755 (39.1)	9609 (51.1)
**Age**, median (interquartile range)	62 (45, 74)	68 (48, 81)	62 (42, 76)	63 (48,74)	61 (47, 71)	57 (44, 68)	54 (41, 66)	59 (40,73)
**Age group, years**								
18–29, n (%)	9436 (10.3)	532 (14.4)	5421 (14.1)	2201 (7.1)	804 (6.3)	320 (7.9)	158 (8.2)	1282 (6.8)
30–39, n (%)	8251 (9)	198 (5.3)	3483 (9.1)	2604 (8.4)	1225 (9.6)	472 (11.7)	269 (13.9)	1966 (10.5)
40–49, n (%)	11,187 (12.2)	250 (6.8)	4039 (10.5)	3963 (12.7)	1839 (14.4)	697 (17.2)	399 (20.7)	2935 (15.6)
50–59, n (%)	14,087 (15.3)	402 (10.9)	5067 (13.2)	5097 (16.4)	2368 (18.5)	787 (19.4)	366 (19)	3521 (18.7)
60–69, n (%)	18,824 (20.5)	614 (16.6)	6854 (17.8)	6945 (22.3)	3016 (23.5)	964 (23.8)	431 (22.3)	4411 (23.5)
70–79, n (%)	16,822 (18.3)	691 (18.7)	6744 (17.5)	6190 (19.9)	2366 (18.5)	588 (14.5)	243 (12.6)	3197 (17.0)
80–89, n (%)	10,995 (11.9)	735 (19.9)	5441 (14.2)	3489 (11.2)	1070 (8.4)	200 (4.9)	60 (3.1)	1330 (7.1)
90–99, n (%)	2379 (2.6)	271 (7.3)	1372 (3.6)	592 (1.9)	121 (0.9)	20 (0.5)	3 (0.2)	144 (0.8)
100+, n (%)	46 (0)	8 (0.2)	25 (0.1)	12 (0)	1 (0)	0 (0)	0 (0)	1 (0)
**CCI level**								
No comorbidity reported	163,554 (67.4)	2163 (58.4)	25580 (66.5)	20292 (65.3)	8409 (65.6)	2725 (67.3)	1279 (66.3)	12,413 (66.1)
Moderate comorbidity	62,875 (25.9)	1213 (32.8)	10373 (27)	9010 (29)	3605 (29.1)	1093 (27)	522 (27.1)	5220 (27.8)
Severe comorbidity	16,208 (6.7)	325 (8.8)	2493 (6.5)	1791 (5.8)	796 (6.2)	230 (5.7)	128 (6.6)	1154 (6.1)
**Year**								
2011, n (%)	14,329 (15.6)	613 (16.6)	5806 (15.1)	4916 (15.8)	2031 (15.9)	661 (16.3)	302 (15.7)	2994 (15.9)
2012, n (%)	22,285 (24.2)	999 (27)	9285 (24.2)	7535 (24.2)	3016 (23.5)	1007 (24.9)	443 (23)	4466 (23.8)
2013, n (%)	26,807 (29.1)	1045 (28.2)	11,322 (29.4)	9068 (29.2)	3733 (29.1)	1120 (27.7)	519 (26.9)	5372 (28.6)
2014, n (%)	28,606 (31.1)	1044 (28.2)	12,033 (31.3)	9574 (30.8)	4030 (31.5)	1260 (31.1)	665 (34.5)	5955 (31.7)
**Number of acute admissions during 2011–2014**								
1, n (%)	50,565 (54.9)	1598 (43.2)	21,394 (55.6)	17,393 (55.9)	6973 (54.4)	2191 (54.1)	1016 (52.7)	10,180 (54.2)
2, n (%)	19,319 (21)	886 (23.9)	7828 (20.4)	6532 (21)	2764 (21.6)	886 (21.9)	423 (21.9)	4073 (21.7)
3, n (%)	8532 (9.3)	435 (11.8)	3511 (9.1)	2772 (8.9)	1227 (9.6)	418 (10.3)	169 (8.8)	1814 (9.7)
4, n (%)	4783 (5.2)	298 (8.1)	1970 (5.1)	1553 (5)	661 (5.2)	192 (4.7)	109 (5.7)	962 (5.1)
5–9, n (%)	7287 (7.9)	392 (10.6)	3136 (8.2)	2353 (7.6)	952 (7.4)	279 (6.9)	175 (9.1)	1406 (7.5)
10–19, n (%)	1401 (1.5)	80 (2.2)	565 (1.5)	444 (1.4)	204 (1.6)	73 (1.8)	35 (1.8)	312 (1.7)
20–29, n (%)	126 (0.1)	10 (0.3)	36 (0.1)	42 (0.1)	27 (0.2)	9 (0.2)	2 (0.1)	38 (0.2)
30+, n (%)	14 (0)	2 (0.1)	6 (0)	4 (0)	2 (0)	0 (0)	0 (0)	2 (0)
**Hospital type**								
Provincial hospital, n (%)	69,301 (75.3)	2747 (74.2)	28,248 (73.5)	23,605 (75.9)	9979 (77.9)	3166 (78.2)	1556 (80.7)	14,701 (78.3)
University hospital, n (%)	22,726 (24.7)	954 (25.8)	10,198 (26.5)	7488 (24.1)	2831 (22.1)	882 (21.8)	373 (19.3)	4086 (21.7)
**Department type**								
Medical, n (%)	37,267 (40.5)	1533 (41.4)	15,069 (39.2)	13,017 (41.9)	5245 (40.9)	1640 (40.5)	763 (39.6)	7648 (40.7)
Acute, n (%)	26,843 (29.2)	1118 (30.2)	10,830 (28.2)	9025 (29.0)	3954 (30.9)	1262 (31.2)	654 (33.9)	5870 (31.2)
Surgical, n (%)	24,799 (26.9)	922 (24.9)	10,968 (28.5)	8186 (26.3)	3259 (25.4)	1015 (25.1)	449 (23.3)	4723 (25.1)
Other, n (%)	3118 (3.4)	128 (3.5)	1579 (4.1)	865 (2.8)	352 (2.7)	131 (3.2)	63 (3.3)	546 (2.9)
**Smoking status**								
Missing, n (%)	37,634 (40.9)	1634 (44.2)	16024 (41.7)	12518 (40.3)	5120 (40.0)	1576 (38.9)	762 (39.5)	7458 (39.7)
Available smoking status	54,393 (59.1)	2067 (55.8)	22422 (58.3)	18575 (59.7)	7690 (60.0)	2472 (61.1)	1167 (60.5)	11,329 (60.3)
Never smoker, n (%)	20,499 (37.7)	540 (26.1)	8521 (38.0)	7065 (38.0)	2876 (37.4)	1013 (41.0)	484 (41.5)	4373 (38.6)
Former smoker, n (%)	16,131 (29.7)	461 (22.3)	5805 (25.9)	6097 (32.8)	2649 (34.4)	753 (29.7)	366 (31.4)	3768 (33.3)
Daily smoker, n (%)	14,738 (27.1)	938 (45.4)	6711 (29.9)	4399 (23.7)	1810 (23.5)	610 (24.7)	270 (23.1)	2690 (23.7)
Occasional smoker, n (%)	3025 (5.6)	128 (6.2)	1385 (6.2)	735 (4.0)	355 (4.6)	96 (3.9)	47 (4.0)	498 (4.4)

### Mortality

**Fig 1:**
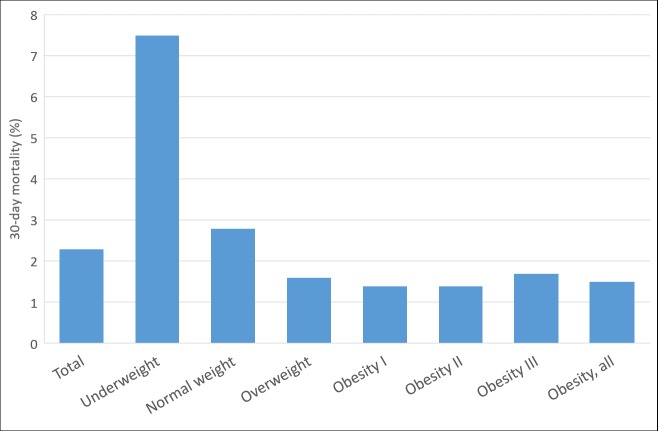
30-day mortality among patients acutely admitted, by BMI category.

Overall 30-day mortality was 2.3%. Compared with normal-weight patients (30-day mortality 2.8%), underweight patients had the highest 30-day mortality (7.5%, aOR: 2.37 (95% CI; 1.95–2.89)) and patients with overweight (aORs: 0.65 (95% CI; 0.56–0.76), and in obesity classes I and II had the lowest 30-day mortality (1.4%, aORs: 0.76 (95% CI; 0.61–0.94), and 0.86 (95% CI; 0.59–1.26), with crude differences diminishing substantially after adjustment for age and smoking differences (Fig 1, Table 2).

**Table 2:** Mortality odds ratios (ORs), using normal weight as reference.

	30-day mortality, %	OR (95% CI)	OR (95% CI) Adjusted for age	OR (95% CI) Adjusted for age and smoking
**BMI <18.5 kg/m**^**2**^	7.5	2.81 (2.45–3.22)	2.17 (1.89–2.51)	2.37 (1.95–2.89)
**BMI 18.5 to 25 kg/m**^**2**^	2.8	Ref.	Ref.	Ref.
**BMI 25 to 30 kg/m**^**2**^	1.6	0.56 (0.51–0.63)	0.61 (0.55–0.68)	0.65 (0.56–0.76)
**BMI 30 to 35 kg/m**^**2**^	1.4	0.50 (0.43–0.58)	0.63 (0.54–0.74)	0.76 (0.61–0.94)
**BMI 35 to 40 kg/m**^**2**^	1.4	0.50 (0.38–0.65)	0.80 (0.61–1.05)	0.86 (0.59–1.26)
**BMI > 40 kg/m**^**2**^	1.7	0.58 (0.41–0.83)	1.12 (0.78–1.60)	1.08 (0.64–1.82)

### Reasons for admission

**Fig 2:**
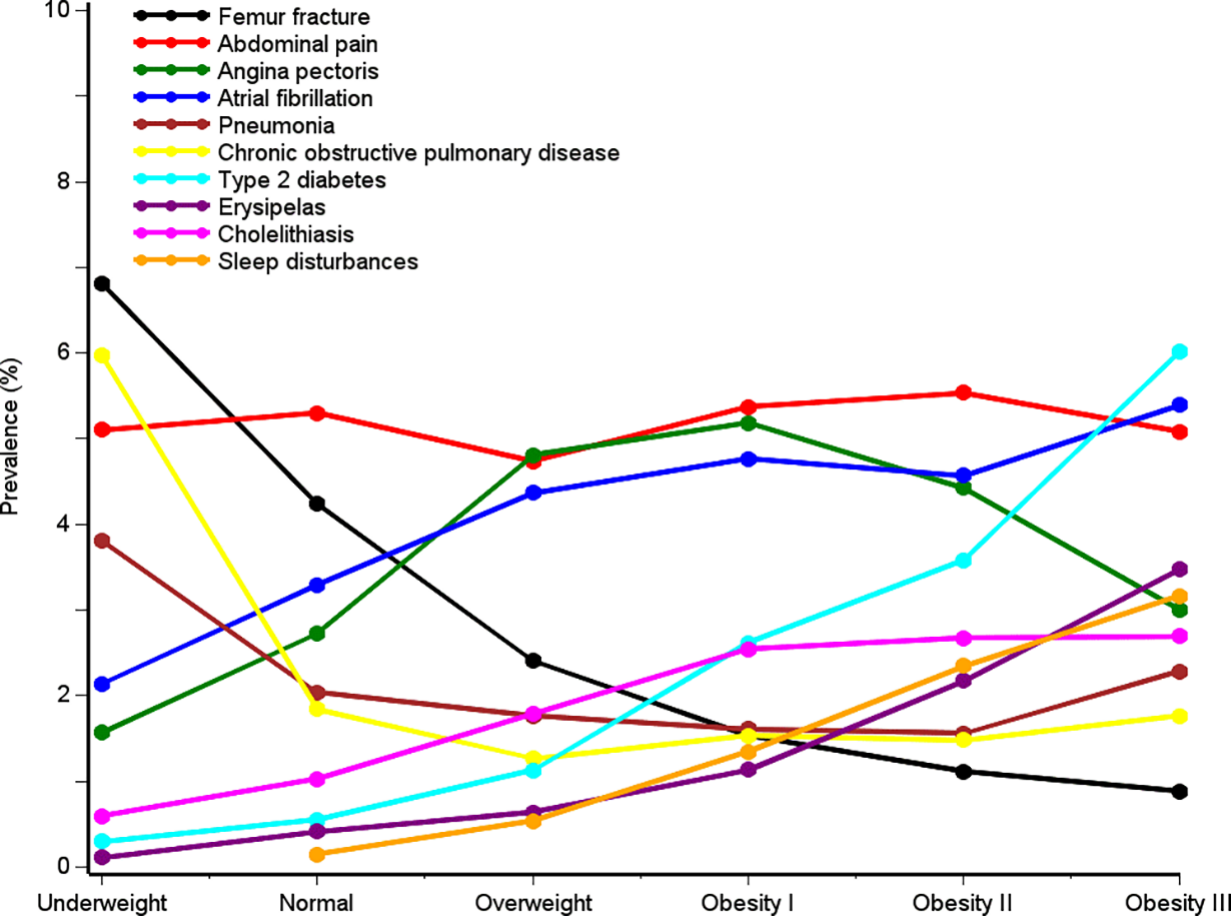
Selected reasons for acute inpatient admission (first-listed diagnosis codes), by BMI category.

Overall, reasons for acute hospital admission differed substantially by BMI range (Fig 2, Tables 3 and 4, and S2 Table).

**Table 3:** Primary discharge diagnosis codes according to ICD-10 chapters.

Disease category	All patients N = 92027	BMI <18.5 kg/m^2^ N = 3701	BMI 18.5 to 25 kg/m^2^ N = 38446	BMI 25 to 30 kg/m^2^ N = 31093	BMI 30 to 35 kg/m^2^ N = 12810	BMI 35 to 40 kg/m^2^ N = 4048	BMI > 40 kg/m^2^ N = 1929
Infectious diseases including pneumonia	3,510 (3.8)	154 (4.2)	1,437 (3.7)	1,090 (3.5)	498 (3.9)	200 (4.9)	131 (6.8)
Neoplasms	7,066 (7.7)	395 (10.7)	3,225 (8.4)	2,317 (7.5)	817 (6.4)	223 (5.5)	89 (4.6)
Hematological diseases	1,364 (1.5)	82 (2.2)	569 (1.5)	413 (1.3)	202 (1.6)	68 (1.7)	30 (1.6)
Endocrine, nutritional and metabolic disorders	4,817 (5.2)	245 (6.6)	1,790 (4.7)	1,384 (4.5)	805 (6.3)	346 (8.5)	247 (12.8)
Mental and behavioral disorders	868 (0.9)	65 (1.8)	413 (1.1)	254 (0.8)	101 (0.8)	26 (0.6)	9 (0.5)
Diseases of the nervous system	5,629 (6.1)	210 (5.7)	2,216 (5.8)	1,910 (6.1)	865 (6.8)	287 (7.1)	141 (7.3)
Diseases of the eye and adnexa	2,520 (2.7)	103 (2.8)	1,024 (2.7)	822 (2.6)	367 (2.9)	129 (3.2)	75 (3.9)
Diseases of the ear and mastoid process	1,527 (1.7)	58 (1.6)	654 (1.7)	538 (1.7)	193 (1.5)	63 (1.6)	21 (1.1)
Diseases of the circulatory system	21,543 (23.4)	536 (14.5)	7,827 (20.4)	8,208 (26.4)	3,487 (27.2)	1,045 (25.8)	440 (22.8)
Diseases of the respiratory system	7,740 (8.4)	645 (17.4)	3,369 (8.8)	2,233 (7.2)	965 (7.5)	337 (8.3)	191 (9.9)
Diseases of the digestive system	11,666 (12.7)	441 (11.9)	4,700 (12.2)	3,975 (12.8)	1,759 (13.7)	527 (13.0)	264 (13.7)
Diseases of the skin and subcutaneous tissue	2,774 (3.0)	65 (1.8)	989 (2.6)	978 (3.1)	466 (3.6)	179 (4.4)	97 (5.0)
Diseases of the musculoskeletal system and connective tissue	6,855 (7.4)	246 (6.6)	2,686 (7.0)	2,366 (7.6)	1,051 (8.2)	355 (8.8)	151 (7.8)
Diseases of the genitourinary system	5,910 (6.4)	219 (5.9)	2,486 (6.5)	1,945 (6.3)	817 (6.4)	288 (7.1)	155 (8.0)
Pregnancy, childbirth and the puerperium	1,319 (1.4)	36 (1.0)	693 (1.8)	331 (1.1)	154 (1.2)	67 (1.7)	38 (2.0)
Conditions originating in the perinatal period	3 (0.0)	0 (0.0)	1 (0.0)	2 (0.0)	0 (0.0)	0 (0.0)	0 (0.0)
Congenital malformations, deformations and chromosomal abnormalities	352 (0.4)	23 (0.6)	167 (0.4)	88 (0.3)	47 (0.4)	21 (0.5)	6 (0.3)
Symptoms, signs and abnormal clinical and laboratory findings	16,272 (17.7)	658 (17.8)	6,857 (17.8)	5,444 (17.5)	2,285 (17.8)	707 (17.5)	321 (16.6)
Injury and poisoning	15,822 (17.2)	646 (17.5)	7,556 (19.7)	4,946 (15.9)	1,860 (14.5)	549 (13.6)	265 (13.7)
External causes of morbidity and mortality	34 (0.0)	4 (0.1)	11 (0.0)	14 (0.0)	4 (0.0)	1 (0.0)	0 (0.0)

**Table 4:** Prevalence ratios (PRs) of discharge diagnosis codes according to ICD-10 chapters.

Disease category	BMI <18.5 kg/m^2^ N = 3701	BMI 18.5 to 25 kg/m^2^ N = 38446	BMI 25 to 30 kg/m^2^ N = 31093	BMI 30 to 35 kg/m^2^ N = 12810	BMI 35 to 40 kg/m^2^ N = 4048	BMI > 40 kg/m^2^ N = 1929
Infectious diseases including pneumonia	1.1 (0.9–1.3)	Ref.	0.9 (0.9–1.0)	1.0 (0.9–1.1)	1.3 (1.1–1.5)	1.8 (1.5–2.2)
Neoplasms	1.3 (1.2–1.4)	Ref.	0.9 (0.8–0.9)	0.8 (0.7–0.8)	0.7 (0.6–0.7)	0.6 (0.4–0.7)
Hematological diseases	1.5 (1.2–1.9)	Ref.	0.9 (0.8–1.0)	1.1 (0.9–1.2)	1.1 (0.9–1.5)	1.1 (0.7–1.5)
Endocrine, nutritional and metabolic disorders	1.4 (1.2–1.6)	Ref.	1.0 (0.9–1.0)	1.3 (1.2–1.5)	1.8 (1.6–2.1)	2.8 (2.4–3.1)
Mental and behavioral disorders	1.6 (1.3–2.1)	Ref.	0.8 (0.7–0.9)	0.7 (0.6–0.9)	0.6 (0.4–0.9)	0.4 (0.2–0.8)
Diseases of the nervous system	1.0 (0.9–1.1)	Ref.	1.1 (1.0–1.1)	1.2 (1.1–1.3)	1.2 (1.1–1.4)	1.3 (1.1–1.5)
Diseases of the eye and adnexa	1.0 (0.9–1.3)	Ref.	1.0 (0.9–1.1)	1.1 (1.0–1.2)	1.2 (1.0–1.4)	1.5 (1.2–1.8)
Diseases of the ear and mastoid process	0.9 (0.7–1.2)	Ref.	1.0 (0.9–1.1)	0.9 (0.8–1.0)	0.9 (0.7–1.2)	0.6 (0.4–1.0)
Diseases of the circulatory system	0.7 (0.7–0.8)	Ref.	1.3 (1.3–1.3)	1.3 (1.3–1.4)	1.3 (1.2–1.3)	1.1 (1.0–1.2)
Diseases of the respiratory system	2.0 (1.8–2.1)	Ref.	0.8 (0.8–0.9)	0.9 (0.8–0.9)	1.0 (0.9–1.1)	1.1 (1.0–1.3)
Diseases of the digestive system	1.0 (0.9–1.1)	Ref.	1.0 (1.0–1.1)	1.1 (1.1–1.2)	1.1 (1.0–1.2)	1.1 (1.0–1.3)
Diseases of the skin and subcutaneous tissue	0.7 (0.5–0.9)	Ref.	1.2 (1.1–1.3)	1.4 (1.3–1.6)	1.7 (1.5–2.0)	2.0 (1.6–2.4)
Diseases of the musculoskeletal system and connective tissue	1.0 (0.8–1.1)	Ref.	1.1 (1.0–1.1)	1.2 (1.1–1.3)	1.3 (1.1–1.4)	1.1 (1.0–1.3)
Diseases of the genitourinary system	0.9 (0.8–1.0)	Ref.	1.0 (0.9–1.0)	1.0 (0.9–1.1)	1.1 (1.0–1.2)	1.2 (1.1–1.5)
Pregnancy, childbirth and the puerperium	0.5 (0.4–0.8)	Ref.	0.6 (0.5–0.7)	0.7 (0.6–0.8)	0.9 (0.7–1.2)	1.1 (0.8–1.5)
Conditions originating in the perinatal period	0.0 (.-.)	Ref.	2.5 (0.2–27.3)	0.0 (.-.)	0.0 (.-.)	0.0 (.-.)
Congenital malformations, deformations and chromosomal abnormalities	1.4 (0.9–2.2)	Ref.	0.7 (0.5–0.8)	0.8 (0.6–1.2)	1.2 (0.8–1.9)	0.7 (0.3–1.6)
Symptoms, signs and abnormal clinical and laboratory findings	1.0 (0.9–1.1)	Ref.	1.0 (1.0–1.0)	1.0 (1.0–1.0)	1.0 (0.9–1.1)	0.9 (0.8–1.0)
Injury and poisoning	0.9 (0.8–1.0)	Ref.	0.8 (0.8–0.8)	0.7 (0.7–0.8)	0.7 (0.6–0.7)	0.7 (0.6–0.8)
External causes of morbidity and mortality	3.8 (1.2–11.9)	Ref.	1.6 (0.7–3.5)	1.1 (0.3–3.4)	0.9 (0.1–6.7)	0.0 (.-.)

Compared with normal-weight individuals as reference (20.4%, Table 2), we observed large differences in the proportion of admissions due to diseases of the circulatory system among patients with underweight: 14.5%, PR: 0.7 (95% CI; 0.7–0.8), overweight: 26.4%, PR: 1.3 (95% CI; 1.3–1.4), and obesity class III: 22.8%, PR: 1.1 (95% CI; 1.0–1.2)). Patients with normal weight had a high frequency of admissions due to injuries (*e*.*g*., fractures) and poisoning (underweight: 17.5%, normal weight: 19.7%, overweight: 15.9%, and obesity class III: 13.7%) (Table 3, see S1 Table for diagnosis codes). For respiratory diseases (*e*.*g*., COPD), we observed a J-shaped association based on visual judgment, with the highest prevalence proportions among patients with underweight (17.4%, PR: 2.0 (95% CI; 1.8–2.1)) and among patients in obesity class III (9.9%, PR: 1.1 (95% CI; 1.0–1.3)) (Tables 3 and 4). Other diseases, including diseases of the digestive system or “symptoms, signs and abnormal clinical findings” had similar frequencies according to BMI category. For several specific conditions within these disease categories, findings were comparable (see Fig 2 and description below).

### Patients with underweight

Patients with underweight differed substantially from patients in the other BMI categories. Median age was high [68 years (IQR: 48–81 years)]; 26.8% were male; many patients smoked daily (45.4%); and 41.6% had previous hospital-diagnosed comorbidities (Table 1). Admissions due to respiratory diseases were more frequent than in the other BMI categories: chronic obstructive pulmonary disease accounted for 6.0% of admissions, pneumonia: 4.5%, pneumonia: 3.8%, bacterial pneumonia for 2.0%, respiratory failure for 1.8%, acute lower respiratory infection for 1.7%, and fracture of the femur for 6.8% (Fig 2, S2 Table).

**Fig 3:**
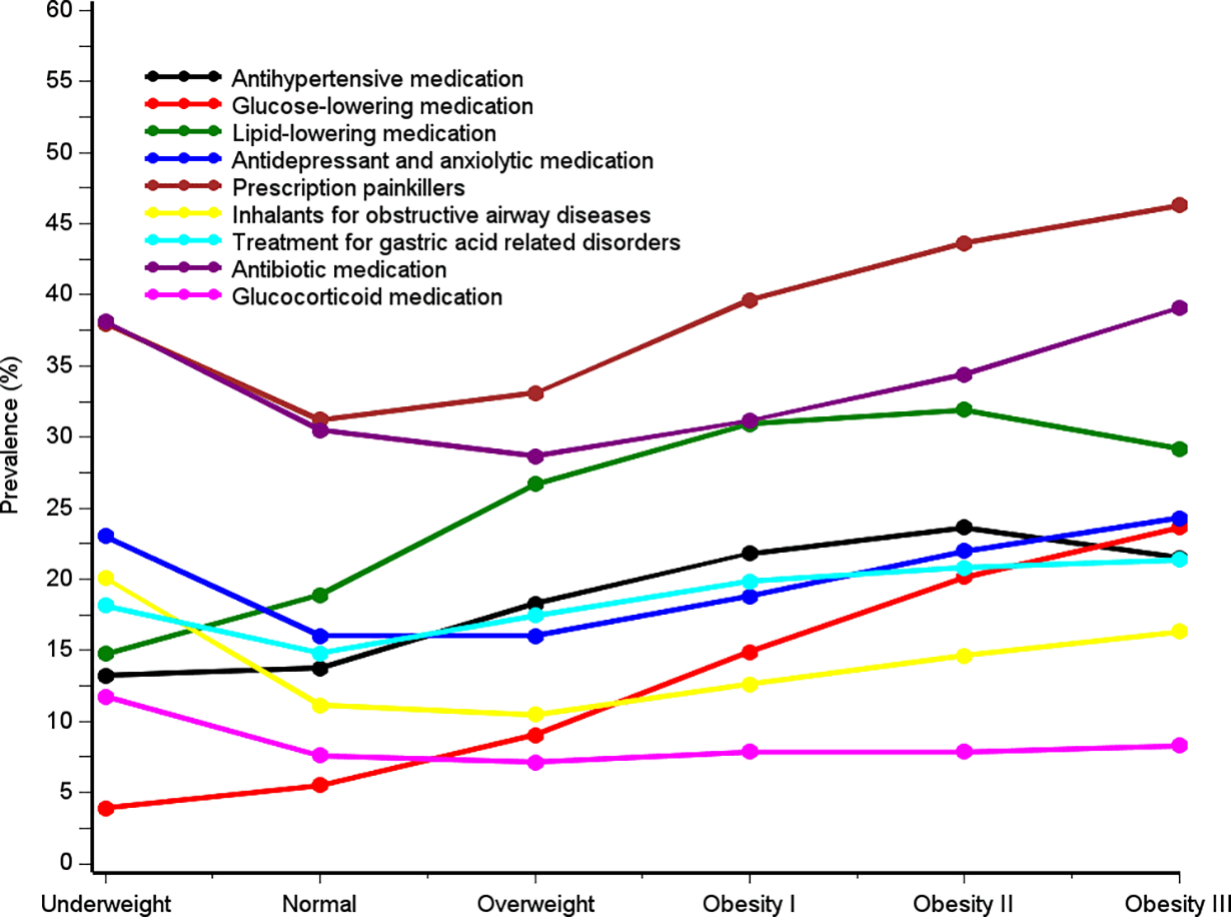
Current use of selected prescription drugs, by BMI category.

Among underweight patients, 38.0% were prescribed painkillers, 38.1% used antibiotic medications, 23.0% used antidepressant and anxiolytic medications; 20.1% used inhalants for obstructive airway diseases, 18.1% used drugs to treat gastric acid-related diseases, and 11.7% used glucocorticoids (Fig 3, S6 Table).

### Patients with normal weight

Among patients with normal weight, the median age was 62 years (IQR: 42–76 years), 45.4% were male, 66.5% had no comorbidities, 29.9% smoked daily, and 30-day mortality was 2.8% (Table 1). The three most common causes of hospital admission were abdominal and pelvic pain (5.3%), fracture of femur (4.2%), and atrial fibrillation and flutter (3.3%) (Fig 2 and S2 Table). Among patients with normal weight, 68.9% were users of any medication (Fig 3, S6 Table).

### Patients with overweight

Among overweight patients, the median age was 63 years (IQR: 48–74 years), 59.2% were male, 65.3% had no comorbidities, 23.7% smoked daily, and 30-day mortality was 1.6% (Table 1). Admission patterns were similar to those of patients with normal weight. However, prevalence of cholelithiasis was higher (1.8%) (Fig 1 and S2 Table). The overall prevalence of medication use was 72.9% (Fig 3, S6 Table). The use of antibiotic medications (28.7%), antidepressants and anxiolytic medications (16.0%), glucocorticoids (10.5%), and inhalants for obstructive airway diseases (10.5%) was lower than in the other BMI categories (Fig 3 and S6 Table).

### Patients with obesity

Patients with BMI ≥30 were relatively young (59 years median), few patients smoked (24%), and 66.1% had no comorbidities.

The median age varied from 61 in obesity class I to 54 years in obesity class III (Table 1). Male gender accounted for 39.1%-54.8% of persons in obesity classes I-III. Thirty-day mortality was lower among patients in obesity classes I and II (1.4%), compared to 1.7% among patients in obesity class III. Patients with obesity had a high overall prevalence of medication use: 78.1% in class I, 81.8% in class II, and 83.6% in class III (Fig 3 and S6 Table). This included a high prevalence of users of glucose-lowering medications (14.9% to 23.6%), antihypertensive medications (21.5% to 21.5%), lipid-lowering medications (30.9% to 29.2%), and antibiotic medications (31.2% to 39.1%). Hospital admissions due to type 2 diabetes increased with higher BMI (obesity class I: 2.6%, obesity class II: 3.6%, and obesity class III: 6.0%) (Fig 3 and S2 Table). Other common causes of hospitalization were cholelithiasis (2.5% to 2.7%), sleep disorders (1.3% to 3.2%), breathing abnormalities (1.3% to 2.4%), erysipelas (1.1% to 3.5%), cutaneous abscess, furuncle, and carbuncle (1.5% to 2.2%) and heart failure (1.6% to 2.0%).

## Discussion

To our knowledge, this is the first study to examine BMI distribution among patients acutely admitted to general hospitals in a setting with universal hospital coverage. This contrasts with previous studies which have examined BMI and mortality either in general populations of adults [3,5] or among patients with specific diseases or inclusion criteria.[6–11]

When comparing our 92,027 hospitalized patients with Denmark’s general population, a recent BMI survey in 2017 found that 2.7% of the Danish population had a self-reported BMI <18.5 kg/m^2^, 47.4% had BMI ≥25 kg/m^2^, and 14.1% had BMI ≥30 kg/m^2^.[35] The corresponding numbers for our hospitalized cohort of 2.4%, 51.0%, and 16.8%, respectively, indicate a slight overrepresentation of patients with high BMI values. This may reflect a truly increased admission rate in underweight or overweight people, or alternatively, more complete BMI recording among hospitalized patients with high BMI.

Overall, we observed considerable differences in patient characteristics and causes of admissions according to BMI category. In line with previous studies on patients with acute admissions,[6–11] we found that patients with underweight had the highest 30-day mortality, while patients in obesity classes I and II had the lowest 30-day mortality, apparently related in part to their lower median age. Compared to patients with normal weight or obesity, patients with underweight were older, and more likely to be female, to have more comorbidities and more abnormal blood test results, and to have a higher prevalence of medication use and smoking. In contrast, patients with obesity were younger, had a lower frequency of smoking and lower use of several types of drugs, including antibiotic drugs and inhalants for obstructive airway diseases, but many more metabolic-syndrome-related risk factors.

It has been suggested that the higher mortality among patients with underweight in previous studies may be explained in part by an association between low BMI, tobacco smoking, and underlying illness leading to non-intentional weight loss. In contrast, our observation of a lower mortality in patients with overweight or obesity as compared with normal weight patients even after controlling for differences in age and smoking seems to confirm the ‘obesity paradox’. [6–11,36] Importantly, we found that patients with normal weight, overweight, and obesity were very similar with regards to both age distribution, comorbidity, and history of ever smoking. Obesity is associated with proinflammatory defenses and increased energy reserves, which may be protective in patients with acute or chronic disease.[37,38] Some studies have reported an age-associated protective effect of overweight on mortality: the higher the age, the more protective is the effect of overweight on mortality.[36] Alternatively, a previous study suggested that a higher proportion of people with obesity may be admitted to hospital, despite similar or lower severity of illness as compared with normal weight individuals.[10] Any lowered admission threshold may cause surveillance bias and lead to underestimation of mortality in obese patients. Another possible explanation on the obesity paradox is the presence of “collider bias”: a situation, where uncorrelated causes [e.g, obesity and infections] “collide” by each competing to trigger the same outcome [e.g. heart failure]–and in the cases where obesity is the cause, the course of the disease may be more mild than in the cases where other causes lead to the disease [eg. infectious cardiomyopathy].[39]

We found a close association between underweight and specific chronic conditions, including chronic pulmonary disease (COPD). Tobacco smoking, [5,26,27] known to have a great impact on disease patterns and mortality, was also more prevalent among underweight persons. These findings corroborate findings in previous cross sectional studies, in which smoking has been related to both underweight [40] and COPD [27] and COPD patients were more underweight than people without COPD.[41] Hospital admissions due to pneumonia also were frequent among patients with underweight, in line with a recent meta-analysis (N = 2,561,839 patients) that reported a relative risk of 1.8 of for community-acquired pneumonia among patients with underweight compared to patients with normal weight.[42] We found that fractures of the femur also were common among patients with underweight corroborating previous findings.[43] Underweight is a documented risk factor for osteoporotic fractures.[44] Although the mechanisms through which low BMI may affect bone mineral density are not completely understood, the low levels of hormones secreted in the adipose tissue, including estrogen, leptin, and interleukin-6, may be protective.[21,45] Smoking is associated with low BMI,[40] low bone mineral density,[46] and fracture risk,[20] and likely contributed to the high number of observed fracture admissions.[21,45]

Corroborating others’ findings, [28] hospital admissions due to type 2 diabetes and use of drugs to treat metabolic syndrome-related diseases were common among patients with overweight and admissions due to type 2 diabetes and use of glucose-lowering drugs increased with increasing BMI (Figs 2 and 3).[28] Cholelithiasis is also associated with obesity and the metabolic syndrome.[31] As seen in Fig 2, our results confirm the well-known association between obesity and obstructive sleep apnea.[47] Also infections including pneumonia and erysipelas increased with increasing BMI (Fig 2), pointing to an association between obesity and skin, wound, respiratory, and other infections, and overall use of antibiotics which is in line with previous studies.[24,25,29,48–51] In contrast to previous findings, we found no clear association between atrial fibrillation and BMI.[23]

The gender distribution differed among the BMI groups: both among patients with underweight and severe obesity, there was a high proportion of females, which may affect the disease risk for e.g. angina pectoris.[52] Further studies are required to investigate the interaction between obesity outcomes and gender in more detail.

### Strengths and limitations

The main strengths of the present study include its unique information on BMI on a very large sample size and the access to complete hospitalization and prescription records.

Our study has several potential limitations. Only 38% of all acutely admitted patients had their BMI measured, and BMI values are likely not missing completely at random. Health care professionals may have an inclination to weigh people with either very low or high BMI compared with patients with normal BMI. Thus, since our data are likely not a random sample of all acutely admitted patients, they cannot be used as an estimation for the BMI distribution of hospital admitted patients in general. However, we present data mostly stratified, and we may argue that within strata of BMI, missingness is at random, and that data per strata (for example, mortality) are not largely invalidated by missingness. Still information bias may have occurred if, theoretically, a larger proportion of critically versus non-critically ill patients with low BMI have their weight measured, while in patients with obesity, both critically and non-critically ill patients may have their BMI measured.

Although inaccurate diagnostic coding may be a concern for the patient characteristics we measured, the physician-coded diagnoses in the DNPR have been reported to be highly valid for many conditions.[14] Concerning prescriptions, the AUPD contains data on prescriptions filled,[19] but no information on actual medication intake. However, several studies have shown good agreement between self-reported medication use or medication use reported by general practitioners and prescriptions filled at pharmacies.[53–55] Finally, data on smoking status relied on information self-reported by patients to their physicians, as recorded in the Central Denmark Region clinical information system at time of admission, and patients may underreport their smoking behavior. However, we assume that this possible underreporting is non-differentially associated with BMI values.

### Conclusion

We have profiled acutely hospitalized patients in Denmark by their BMI. We documented a high short-term mortality among patients with underweight, however, we found evidence that clinical characteristics differ substantially by BMI category in hospitalized patients. Patients with underweight have a notably high frequency of smoking, admissions due to respiratory diseases, osteoporotic fractures, and comorbidity. Patients with obesity are often hospitalized due to skin infections, cholelithiasis, sleep disorders, diabetes, and heart failure. These data may prove valuable for future studies on the prognostic effect of BMI following hospital admission for a range of diseases.

## Supporting information

S1 Table Diagnosis codes for selected diseases and according to ICD-10 chapters used for disease categories. (DOCX)

S2 Table The 15 most common ICD-10 diagnoses at the three-digit level in the Danish National Patient Registry, according to body mass index category. (DOCX)

S3 Table ICD-10 codes for conditions included in the Charlson Comorbidity Index. (DOCX)

S4 Table Prevalence of comorbidities in the Charlson Comorbidity Score, by BMI category. (DOCX)

S5 Table Anatomical Therapeutic Chemical Classification System codes (ATC-codes) for prescription medications from the Danish National Prescription Health Service Database. (DOCX)

S6 Table Prevalences of prehospital medication users in the cohort according to BMI category. (DOCX)

## References

[1] Prospective Studies Collaboration, Whitlock G, Lewington S, Sherliker P, Clarke R, Emberson J, et al Body-mass index and cause-specific mortality in 900 000 adults: collaborative analyses of 57 prospective studies. Lancet 2009 3 28;373(9669):1083–1096. doi: 10.1016/S0140-6736(09)60318-4 19299006 10.1016/S0140-6736(09)60318-4 PMC2662372

[2] Berrington de Gonzalez A, Hartge P, Cerhan JR, Flint AJ, Hannan L, MacInnis RJ, et al Body-mass index and mortality among 1.46 million white adults. N Engl J Med 2010 12 2;363(23):2211–2219. doi: 10.1056/NEJMoa1000367 21121834 10.1056/NEJMoa1000367 PMC3066051

[3] Flegal KM, Kit BK, Orpana H, Graubard BI. Association of all-cause mortality with overweight and obesity using standard body mass index categories: a systematic review and meta-analysis. JAMA 2013 1 2;309(1):71–82. doi: 10.1001/jama.2012.113905 23280227 10.1001/jama.2012.113905 PMC4855514

[4] American College of Cardiology/American Heart Association Task Force on Practice Guidelines, Obesity Expert Panel, 2013. Expert Panel Report: Guidelines (2013) for the management of overweight and obesity in adults. Obesity (Silver Spring) 2014 7;22 Suppl 2:S41–410. 24227637 10.1002/oby.20660

[5] Global BMI Mortality Collaboration. Body-mass index and all-cause mortality: individual-participant-data meta-analysis of 239 prospective studies in four continents. Lancet 2016 8 20;388(10046):776–786. doi: 10.1016/S0140-6736(16)30175-1 27423262 10.1016/S0140-6736(16)30175-1 PMC4995441

[6] Doehner W, Clark A, Anker SD. The obesity paradox: weighing the benefit. Eur Heart J 2010 1;31(2):146–148. doi: 10.1093/eurheartj/ehp339 19734553 10.1093/eurheartj/ehp339

[7] Oreopoulos A, Padwal R, Kalantar-Zadeh K, Fonarow GC, Norris CM, McAlister FA. Body mass index and mortality in heart failure: a meta-analysis. Am Heart J 2008 7;156(1):13–22. doi: 10.1016/j.ahj.2008.02.014 18585492 10.1016/j.ahj.2008.02.014

[8] Prieto-Alhambra D, Premaor MO, Aviles FF, Castro AS, Javaid MK, Nogues X, et al Relationship between mortality and BMI after fracture: a population-based study of men and women aged >/ = 40 years. J Bone Miner Res 2014 8;29(8):1737–1744. doi: 10.1002/jbmr.2209 24615695 10.1002/jbmr.2209

[9] Romero-Corral A, Montori VM, Somers VK, Korinek J, Thomas RJ, Allison TG, et al Association of bodyweight with total mortality and with cardiovascular events in coronary artery disease: a systematic review of cohort studies. Lancet 2006 8 19;368(9536):666–678. doi: 10.1016/S0140-6736(06)69251-9 16920472 10.1016/S0140-6736(06)69251-9

[10] Sakr Y, Alhussami I, Nanchal R, Wunderink RG, Pellis T, Wittebole X, et al Being Overweight Is Associated With Greater Survival in ICU Patients: Results From the Intensive Care Over Nations Audit. Crit Care Med 2015 12;43(12):2623–2632. doi: 10.1097/CCM.0000000000001310 26427591 10.1097/CCM.0000000000001310

[11] Barba R, Marco J, Ruiz J, Canora J, Hinojosa J, Plaza S, et al The obesity paradox in stroke: impact on mortality and short-term readmission. J Stroke Cerebrovasc Dis 2015 4;24(4):766–770. doi: 10.1016/j.jstrokecerebrovasdis.2014.11.002 25670014 10.1016/j.jstrokecerebrovasdis.2014.11.002

[12] Aune D, Sen A, Prasad M, Norat T, Janszky I, Tonstad S, et al BMI and all cause mortality: systematic review and non-linear dose-response meta-analysis of 230 cohort studies with 3.74 million deaths among 30.3 million participants. BMJ 2016 5 4;353:i2156 doi: 10.1136/bmj.i2156 27146380 10.1136/bmj.i2156 PMC4856854

[13] World cancer Research Fund/American Institute for Cancer Research. Food, Nutrtion, Physical Activity, and the Prevention of Cancer. A Global Perspective. Washington, DC: AICR 2007.

[14] Schmidt M, Schmidt SA, Sandegaard JL, Ehrenstein V, Pedersen L, Sorensen HT. The Danish National Patient Registry: a review of content, data quality, and research potential. Clin Epidemiol 2015 11 17;7:449–490. doi: 10.2147/CLEP.S91125 26604824 10.2147/CLEP.S91125 PMC4655913

[15] Gho JMIH, Schmidt AF, Pasea L, Koudstaal S, Pujades-Rodriguez M, Denaxas S, et al An electronic health records cohort study on heart failure following myocardial infarction in England: incidence and predictors. BMJ Open 2018 3 3;8(3):e018331-2017-018331. 10.1136/bmjopen-2017-018331 PMC5855447 29502083

[16] Appari A, Eric Johnson M, Anthony DL. Meaningful use of electronic health record systems and process quality of care: evidence from a panel data analysis of U.S. acute-care hospitals. Health Serv Res 2013 4;48(2 Pt 1):354–375. 22816527 10.1111/j.1475-6773.2012.01448.x PMC3626353

[17] Jha AK, DesRoches CM, Campbell EG, Donelan K, Rao SR, Ferris TG, et al Use of electronic health records in U.S. hospitals. N Engl J Med 2009 4 16;360(16):1628–1638. doi: 10.1056/NEJMsa0900592 19321858 10.1056/NEJMsa0900592

[18] Schmidt M, Pedersen L, Sorensen HT. The Danish Civil Registration System as a tool in epidemiology. Eur J Epidemiol 2014 8;29(8):541–549. doi: 10.1007/s10654-014-9930-3 24965263 10.1007/s10654-014-9930-3

[19] Ehrenstein V, Antonsen S, Pedersen L. Existing data sources for clinical epidemiology: Aarhus University Prescription Database. Clin Epidemiol 2010 12 2;2:273–279. doi: 10.2147/CLEP.S13458 21152254 10.2147/CLEP.S13458 PMC2998817

[20] Shen GS, Li Y, Zhao G, Zhou HB, Xie ZG, Xu W, et al Cigarette smoking and risk of hip fracture in women: a meta-analysis of prospective cohort studies. Injury 2015 7;46(7):1333–1340. doi: 10.1016/j.injury.2015.04.008 25956674 10.1016/j.injury.2015.04.008

[21] Yang S, Center JR, Eisman JA, Nguyen TV. Association between fat mass, lean mass, and bone loss: the Dubbo Osteoporosis Epidemiology Study. Osteoporos Int 2015 4;26(4):1381–1386. doi: 10.1007/s00198-014-3009-6 25572048 10.1007/s00198-014-3009-6

[22] Dicker D, Feldman BS, Leventer-Roberts M, Benis A. Obesity or smoking: Which factor contributes more to the incidence of myocardial infarction? Eur J Intern Med 2016 7;32:43–46. doi: 10.1016/j.ejim.2016.03.029 27151319 10.1016/j.ejim.2016.03.029

[23] Lau DH, Nattel S, Kalman JM, Sanders P. Modifiable Risk Factors and Atrial Fibrillation. Circulation 2017 8 8;136(6):583–596. doi: 10.1161/CIRCULATIONAHA.116.023163 28784826 10.1161/CIRCULATIONAHA.116.023163

[24] Kornum JB, Norgaard M, Dethlefsen C, Due KM, Thomsen RW, Tjonneland A, et al Obesity and risk of subsequent hospitalisation with pneumonia. Eur Respir J 2010 12;36(6):1330–1336. doi: 10.1183/09031936.00184209 20351023 10.1183/09031936.00184209

[25] Fisher-Hoch SP, Mathews CE, McCormick JB. Obesity, diabetes and pneumonia: the menacing interface of non-communicable and infectious diseases. Trop Med Int Health 2013 12;18(12):1510–1519. doi: 10.1111/tmi.12206 24237786 10.1111/tmi.12206

[26] Liu Y, Pleasants RA, Croft JB, Lugogo N, Ohar J, Heidari K, et al Body mass index, respiratory conditions, asthma, and chronic obstructive pulmonary disease. Respir Med 2015 7;109(7):851–859. doi: 10.1016/j.rmed.2015.05.006 26006753 10.1016/j.rmed.2015.05.006 PMC4487766

[27] Halldin CN, Doney BC, Hnizdo E. Changes in prevalence of chronic obstructive pulmonary disease and asthma in the US population and associated risk factors. Chron Respir Dis 2015 2;12(1):47–60. doi: 10.1177/1479972314562409 25540134 10.1177/1479972314562409 PMC5588663

[28] Must A, Spadano J, Coakley EH, Field AE, Colditz G, Dietz WH. The disease burden associated with overweight and obesity. JAMA 1999 10 27;282(16):1523–1529. 10546691 10.1001/jama.282.16.1523

[29] Falagas ME, Kompoti M. Obesity and infection. Lancet Infect Dis 2006 7;6(7):438–446. doi: 10.1016/S1473-3099(06)70523-0 16790384 10.1016/S1473-3099(06)70523-0

[30] Krasagakis K, Samonis G, Valachis A, Maniatakis P, Evangelou G, Tosca A. Local complications of erysipelas: a study of associated risk factors. Clin Exp Dermatol 2011 6;36(4):351–354. doi: 10.1111/j.1365-2230.2010.03978.x 21198795 10.1111/j.1365-2230.2010.03978.x

[31] Portincasa P, Moschetta A, Palasciano G. Cholesterol gallstone disease. Lancet 2006 7 15;368(9531):230–239. doi: 10.1016/S0140-6736(06)69044-2 16844493 10.1016/S0140-6736(06)69044-2

[32] Ng SS, Chan RS, Woo J, Chan TO, Cheung BH, Sea MM, et al A Randomized Controlled Study to Examine the Effect of a Lifestyle Modification Program in OSA. Chest 2015 11;148(5):1193–1203. doi: 10.1378/chest.14-3016 25763792 10.1378/chest.14-3016

[33] Thygesen SK, Christiansen CF, Christensen S, Lash TL, Sorensen HT. The predictive value of ICD-10 diagnostic coding used to assess Charlson comorbidity index conditions in the population-based Danish National Registry of Patients. BMC Med Res Methodol 2011 5 28;11:83-2288-11-83. 10.1186/1471-2288-11-83 PMC3125388 21619668

[34] Benchimol EI, Smeeth L, Guttmann A, Harron K, Moher D, Petersen I, et al The REporting of studies Conducted using Observational Routinely-collected health Data (RECORD) statement. PLoS Med 2015 10 6;12(10):e1001885 doi: 10.1371/journal.pmed.1001885 26440803 10.1371/journal.pmed.1001885 PMC4595218

[35] The Danish Health and Medicines Authorities, National Institute of Public Health. Danskernes Sundhed. Tal fra Den Nationale Sundhedsprofil. 2018; Available at: http://www.danskernessundhed.dk/, 2018.

[36] Afzal S, Tybjaerg-Hansen A, Jensen GB, Nordestgaard BG. Change in Body Mass Index Associated With Lowest Mortality in Denmark, 1976–2013. JAMA 2016 5 10;315(18):1989–1996. doi: 10.1001/jama.2016.4666 27163987 10.1001/jama.2016.4666

[37] Ridker PM, Everett BM, Thuren T, MacFadyen JG, Chang WH, Ballantyne C, et al Antiinflammatory Therapy with Canakinumab for Atherosclerotic Disease. N Engl J Med 2017 9 21;377(12):1119–1131. doi: 10.1056/NEJMoa1707914 28845751 10.1056/NEJMoa1707914

[38] Roth J. Evolutionary speculation about tuberculosis and the metabolic and inflammatory processes of obesity. JAMA 2009 6 24;301(24):2586–2588. doi: 10.1001/jama.2009.930 19549976 10.1001/jama.2009.930

[39] Stovitz SD, Banack HR, Kaufman JS. Structural Bias in Studies of Cardiovascular Disease: Let's Not Be Fooled by the "Obesity Paradox". Can J Cardiol 2017 11 7. 10.1016/j.cjca.2017.10.025 29289401

[40] Wang Q. Smoking and body weight: evidence from China health and nutrition survey. BMC Public Health 2015 12 14;15:1238-015-2549-9. 10.1186/s12889-015-2549-9 PMC4678710 26666320

[41] Stewart Coats AJ, Shewan LG. A comparison of research into cachexia, wasting and related skeletal muscle syndromes in three chronic disease areas. Int J Cardiol 2017 5 15;235:33–36. doi: 10.1016/j.ijcard.2017.02.136 28291621 10.1016/j.ijcard.2017.02.136

[42] Phung DT, Wang Z, Rutherford S, Huang C, Chu C. Body mass index and risk of pneumonia: a systematic review and meta-analysis. Obes Rev 2013 10;14(10):839–857. doi: 10.1111/obr.12055 23800284 10.1111/obr.12055

[43] Prieto-Alhambra D, Pages-Castella A, Wallace G, Javaid MK, Judge A, Nogues X, et al Predictors of fracture while on treatment with oral bisphosphonates: a population-based cohort study. J Bone Miner Res 2014 1;29(1):268–274. doi: 10.1002/jbmr.2011 23761350 10.1002/jbmr.2011 PMC3867340

[44] Compston JE, Flahive J, Hosmer DW, Watts NB, Siris ES, Silverman S, et al Relationship of weight, height, and body mass index with fracture risk at different sites in postmenopausal women: the Global Longitudinal study of Osteoporosis in Women (GLOW). J Bone Miner Res 2014 2;29(2):487–493. doi: 10.1002/jbmr.2051 23873741 10.1002/jbmr.2051 PMC4878680

[45] Shapses SA, Riedt CS. Bone, body weight, and weight reduction: what are the concerns? J Nutr 2006 6;136(6):1453–1456. doi: 10.1093/jn/136.6.1453 16702302 10.1093/jn/136.6.1453 PMC4016235

[46] Jaramillo JD, Wilson C, Stinson DS, Lynch DA, Bowler RP, Lutz S, et al Reduced Bone Density and Vertebral Fractures in Smokers. Men and COPD Patients at Increased Risk. Ann Am Thorac Soc 2015 5;12(5):648–656. doi: 10.1513/AnnalsATS.201412-591OC 25719895 10.1513/AnnalsATS.201412-591OC PMC4418341

[47] Zeng F, Wang X, Hu W, Wang L. Association of adiponectin level and obstructive sleep apnea prevalence in obese subjects. Medicine (Baltimore) 2017 8;96(32):e7784. 28796077 10.1097/MD.0000000000007784 PMC5556243

[48] Kwong JC, Campitelli MA, Rosella LC. Obesity and respiratory hospitalizations during influenza seasons in Ontario, Canada: a cohort study. Clin Infect Dis 2011 9;53(5):413–421. doi: 10.1093/cid/cir442 21844024 10.1093/cid/cir442 PMC3156143

[49] Kaspersen KA, Pedersen OB, Petersen MS, Hjalgrim H, Rostgaard K, Moller BK, et al Obesity and risk of infection: results from the Danish Blood Donor Study. Epidemiology 2015 7;26(4):580–589. doi: 10.1097/EDE.0000000000000301 25978794 10.1097/EDE.0000000000000301

[50] Leth RA, Moller JK, Thomsen RW, Uldbjerg N, Norgaard M. Risk of selected postpartum infections after cesarean section compared with vaginal birth: a five-year cohort study of 32,468 women. Acta Obstet Gynecol Scand 2009 9;88(9):976–983. doi: 10.1080/00016340903147405 19642043 10.1080/00016340903147405

[51] Harpsoe MC, Nielsen NM, Friis-Moller N, Andersson M, Wohlfahrt J, Linneberg A, et al Body Mass Index and Risk of Infections Among Women in the Danish National Birth Cohort. Am J Epidemiol 2016 6 1;183(11):1008–1017. doi: 10.1093/aje/kwv300 27188940 10.1093/aje/kwv300

[52] Schmidt M, Horvath-Puho E, Pedersen L, Sorensen HT, Botker HE. Time-dependent effect of preinfarction angina pectoris and intermittent claudication on mortality following myocardial infarction: A Danish nationwide cohort study. Int J Cardiol 2015;187:462–469. doi: 10.1016/j.ijcard.2015.03.328 25846654 10.1016/j.ijcard.2015.03.328

[53] Wogelius P, Poulsen S, Sorensen HT. Validity of parental-reported questionnaire data on Danish children's use of asthma-drugs: a comparison with a population-based prescription database. Eur J Epidemiol 2005;20(1):17–22. 15756900 10.1007/s10654-004-1501-6

[54] Lokkegaard EL, Johnsen SP, Heitmann BL, Stahlberg C, Pedersen AT, Obel EB, et al The validity of self-reported use of hormone replacement therapy among Danish nurses. Acta Obstet Gynecol Scand 2004 5;83(5):476–481. doi: 10.1111/j.0001-6349.2004.00376.x 15059162 10.1111/j.0001-6349.2004.00376.x

[55] Johannesdottir SA, Maegbaek ML, Hansen JG, Lash TL, Pedersen L, Ehrenstein V. Correspondence between general practitioner-reported medication use and timing of prescription dispensation. Clin Epidemiol 2012;4:13–18. doi: 10.2147/CLEP.S26958 22291479 10.2147/CLEP.S26958 PMC3266865

