# Supplementary material for: Body Mass Index of 92,027 patients acutely admitted to general hospitals in Denmark: Associated clinical characteristics and 30-day mortality
Source: PLoS One. 2018 Apr 16;13(4):e0195853. doi: 10.1371/journal.pone.0195853 (PMC5901987; doi:10.1371/journal.pone.0195853)
Supplement: S1 Table — (DOCX) [file pone.0195853.s001.docx]

**S1 Table. Diagnosis codes for selected diseases and according to ICD-10 chapters used for disease categories.**

| **Disease category** | **ICD-10 codes** |
| --- | --- |
| **Selected diseases** |  |
| Femur fracture | S72 |
| Abdominal pain | R10 |
| Angina | I20 |
| Atrial fibrillation | I48 |
| Pneumonia | J18 |
| Chronic obstructive pulmonary disease | J44 |
| Type 2 diabetes | E11 |
| Erysipelas | A46 |
| Cholelithiasis | K80 |
| Sleep disturbances | G47 |
| **ICD-10 chapters** |  |
| Infectious diseases including pneumonia | A, B |
| Neoplasms | C, D0-D4 |
| Hematological diseases | D5-D8 |
| Endocrine, nutritional and metabolic disorders | E |
| Mental and behavioral disorders | F |
| Diseases of the nervous system | G |
| Diseases of the eye and adnexa | H0-H5 |
| Diseases of the ear and mastoid process | H6-H9 |
| Diseases of the circulatory system | I |
| Diseases of the respiratory system | J |
| Diseases of the digestive system | K |
| Diseases of the skin and subcutaneous tissue | L |
| Diseases of the musculoskeletal system and connective tissue | M |
| Diseases of the genitourinary system | N |
| Pregnancy, childbirth and the puerperium | O |
| Conditions originating in the perinatal period | P |
| Congenital malformations, deformations and chromosomal abnormalities | Q |
| Symptoms, signs and abnormal clinical and laboratory findings | R |
| Injury and poisoning | S, T |
| External causes of morbidity and mortality | X, Y |
