# Supplementary material for: Body Mass Index of 92,027 patients acutely admitted to general hospitals in Denmark: Associated clinical characteristics and 30-day mortality
Source: PLoS One. 2018 Apr 16;13(4):e0195853. doi: 10.1371/journal.pone.0195853 (PMC5901987; doi:10.1371/journal.pone.0195853)
Supplement: S2 Table — (DOCX) [file pone.0195853.s002.docx]

**S2 Table. The 15 most common ICD-10 diagnoses at the three-digit level in the Danish National Patient Registry, according to body mass index category, 2011-2014. ^a^
Note: ^a^Only primary diagnoses are included.**

| **Total** | | **BMI<18.5 kg/m^2^** | | **BMI 18.5 to <25 kg/m^2^** | | **BMI 25 to 30 kg/m^2^** |  |
| --- | --- | --- | --- | --- | --- | --- | --- |
| **Diagnosis (ICD-10 code)** | **n (%)** | **Diagnosis (ICD-10 code)** | **n (%)** | **Diagnosis (ICD-10 code)** | **n (%)** | **Diagnosis (ICD-10 code)** | **n (%)** |
| Any | 92027 (100) | Any | 3701 (100) | Any | 38446 (100) | Any | 31093 (100) |
| 1. Abdominal and pelvic pain (R10) | 4709 (5.1) | 1. Fracture of femur (S72) | 252 (6.8) | 1. Abdominal and pelvic pain (R10) | 2038 (5.3) | 1. Angina pectoris (I20) | 1495 (4.8) |
| 2. Atrial fibrillation and flutter (I48) | 3601 (3.9) | 2. Other chronic obstructive pulmonary disease (J44) | 221 (6.0) | 2. Fracture of femur (S72) | 1630 (4.2) | 2. Abdominal and pelvic pain (R10) | 1472 (4.7) |
| 3. Angina pectoris (I20) | 3502 (3.8) | 3. Abdominal and pelvic pain (R10) | 189 (5.1) | 3. Atrial fibrillation and flutter (I48) | 1265 (3.3) | 3. Atrial fibrillation and flutter (I48) | 1358 (4.4) |
| 4. Cerebral infarction (I63) | 3106 (3.4) | 4. Pneumonia, organism unspecified (J18) | 141 (3.8) | 4. Cerebral infarction (I63) | 1208 (3.1) | 4. Cerebral infarction (I63) | 1222 (3.9) |
| 5. Fracture of femur (S72) | 2890 (3.1) | 5.Atrial fibrillation and flutter (I48) | 79 (2.1) | 5. Angina pectoris (I20) | 1048 (2.7) | 5. Acute myocardial infarction (I21) | 1164 (3.7) |
| 6. Acute myocardial infarction (I21) | 2769 (3.0) | 6. Bacterial pneumonia, not elsewhere classified (J15) | 75 (2.0) | 6. Acute myocardial infarction (I21) | 967 (2.5) | 6. Fracture of femur (S72) | 749 (2.4) |
| 7. Fracture of lower leg, including ankle (S82) | 1823 (2.0) | 7. Cerebral infarction (I63) | 73 (2.0) | 7. Fracture of forearm (S52) | 841 (2.2) | 7. Transient cerebral ischaemic attacks (G45) | 670 (2.2) |
| 8. Pneumonia, organism unspecified (J18) | 1784 (1.9) | 8. Syncope and collapse (R55) | 70 (1.9) | 8. Syncope and collapse (R55) | 799 (2.1) | 8. Fracture of lower leg, including ankle (S82) | 650 (2.1) |
| 9. Acute appendicitis (K35) | 1771 (1.9) | 9. Respiratory failure, not elsewhere classified (J96) | 66 (1.8) | 9. Pneumonia, organism unspecified (J18) | 782 (2.0) | 9. Acute appendicitis (K35) | 637 (2.0) |
| 10. Syncope and collapse (R55) | 1718 (1.9) | 10. Volume depletion (E86) | 65 (1.8) | 10. Acute appendicitis (K35) | 782 (2.0) | 10. Pain in throat and chest (R07) | 623 (2.0) |
| 11. Fracture of forearm (S52) | 1656 (1.8) | 11. Unspecified acute lower respiratory infection (J22) | 62 (1.7) | 11. Other chronic obstructive pulmonary disease (J44) | 707 (1.8) | 11. Syncope and collapse (R55) | 599 (1.9) |
| 12. Pain in throat and chest (R07) | 1629 (1.8) | 12. Angina pectoris (I20) | 58 (1.6) | 12. Fracture of lower leg, including ankle (S82) | 707 (1.8) | 12. Cholelithiasis (K80) | 556 (1.8) |
| 13. Transient cerebral ischaemic attacks (G45) | 1615 (1.8) | 13. Fracture of forearm (S52) | 58 (1.6) | 13. Transient cerebral ischaemic attacks G45) | 620 (1.6) | 13. Pneumonia, organism unspecified (J18) | 548 (1.8) |
| 15. Other chronic obstructive pulmonary disease (J44) | 1612 (1.8) | 14. Other functional intestinal disorders (K59) | 57 (1.5) | 14. Pain in throat and chest (R07) | 584 (1.5) | 14. Fracture of forearm (S52) | 499 (1.6) |
| 15. Cholelithiasis (K80) | 1459 (1.6) | 15. Acute myocardial infarction (I21) | 50 (1.4) | 15. Nonrheumatic aortic valve disorders (I35) | 464 (1.2) | 15. Nonrheumatic aortic valve disorders (I35) | 468 (1.5) |

**S2 Appendix. The 15 most common ICD-10 diagnoses at the three-digit level in the Danish National Patient Registry, according to body mass index category, 2011-2014.^a^
Note: ^a^Only primary diagnoses are included.**

| **BMI 30 to 35 kg/m^2^** | | **BMI 35 to 40 kg/m^2^** | | **BMI > 40 kg/m^2^** | |
| --- | --- | --- | --- | --- | --- |
| **Diagnosis (ICD-10 code)** | **n (%)** | **Diagnosis (ICD-10 code)** | **n (%)** | **Diagnosis (ICD-10 code)** | **n (%)** |
| Any | 12810 (100) | Any | 4048 (100) | Any | 1929 (100) |
| 1. Abdominal and pelvic pain (R10) | 688 (5.4) | 1. Abdominal and pelvic pain (R10) | 224 (5.5) | 1. Type 2 diabetes mellitus (E11) | 116 (6.0) |
| 2. Angina pectoris (I20) | 664 (5.2) | 2. Atrial fibrillation and flutter (I48) | 185 (4.6) | 2. Atrial fibrillation and flutter (I48) | 104 (5.4) |
| 3. Atrial fibrillation and flutter (I48) | 610 (4.8) | 3. Angina pectoris (I20) | 179 (4.4) | 3. Abdominal and pelvic pain (R10) | 98 (5.1) |
| 4. Cerebral infarction (I63) | 439 (3.4) | 4. Type 2 diabetes mellitus (E11) | 145 (3.6) | 4. Obesity (E66) | 73 (3.8) |
| 5. Acute myocardial infarction (I21) | 425 (3.3) | 5. Cerebral infarction (I63) | 127 (3.1) | 5. Erysipelas (A46) | 67 (3.5) |
| 6. Type 2 diabetes mellitus (E11) | 335 (2.6) | 6. Acute myocardial infarction (I21) | 119 (2.9) | 6. Sleep disorders (G47) | 61 (3.2) |
| 7. Cholelithiasis (K80) | 326 (2.5) | 7. Cholelithiasis (K80) | 108 (2.7) | 7. Angina pectoris (I20) | 58 (3.0) |
| 8. Fracture of lower leg, including ankle (S82) | 292 (2.3) | 8. Fracture of lower leg, including ankle (S82) | 104 (2.6) | 8. Cholelithiasis (K80) | 52 (2.7) |
| 9. Pain in throat and chest (R07) | 265 (2.1) | 9. Sleep disorders (G47) | 95 (2.3) | 9. Abnormalities of breathing (R06) | 47 (2.4) |
| 10. Transient cerebral ischaemic attacks (G45) | 227 (1.8) | 10. Erysipelas (A46) | 88 (2.2) | 10. Acute myocardial infarction (I21) | 44 (2.3) |
| 11. Essential hypertension (I10) | 225 (1.8) | 11. Abnormalities of breathing (R06) | 84 (2.1) | 11. Pneumonia, organism unspecified (J18) | 44 (2.3) |
| 12. Acute appendicitis (K35) | 218 (1.7) | 12. Pain in throat and chest (R07) | 82 (2.0) | 12. Pain in throat and chest (R07) | 44 (2.3) |
| 13. Pneumonia, organism unspecified (J18) | 206 (1.6) | 13. Essential hypertension (I10) | 78 (1.9) | 13. Cutaneous abscess, furuncle and carbuncle (L02) | 43 (2.2) |
| 15. Heart failure (I50) | 204 (1.6) | 14. Heart failure (I50) | 75 (1.9) | 14. Fracture of lower leg, including ankle (S82) | 40 (2.1) |
| 15. Fracture of femur (S72) | 197 (1.5) | 15. Cutaneous abscess, furuncle and carbuncle (L02) | 68 (1.7) | 15. Heart failure (I50) | 39 (2.0) |
