# Supplementary material for: Body Mass Index of 92,027 patients acutely admitted to general hospitals in Denmark: Associated clinical characteristics and 30-day mortality
Source: PLoS One. 2018 Apr 16;13(4):e0195853. doi: 10.1371/journal.pone.0195853 (PMC5901987; doi:10.1371/journal.pone.0195853)
Supplement: S3 Table — (DOCX) [file pone.0195853.s003.docx]

**S3 Table. ICD-10 codes for conditions included in the Charlson Comorbidity Index.**

| **Disease** | **IDC-10** | **Score** |
| --- | --- | --- |
| **Myocardial infarction** | I21; I22; I23 | 1 |
| **Congestive heart failure** | I50; I11.0; I13.0; I13.2 | 1 |
| **Peripheral vascular disease** | I70; I71; I72; I73; I74; I77 | 1 |
| **Cerebrovascular disease** | I60–I69; G45; G46 | 1 |
| **Dementia** | F00–F03; F05.1; G30 | 1 |
| **Chronic pulmonary disease** | J40–J47; J60–J67; J68.4; J70.1; J70.3; J84.1; J92.0; J96.1; J98.2; J98.3 | 1 |
| **Connective tissue disease** | M05; M06; M08; M09;M30;M31; M32; M33; M34; M35; M36; D86 | 1 |
| **Ulcer disease** | K22.1; K25-K28 | 1 |
| **Mild liver disease** | B18; K70.0-K70.3; K70.9; K71; K73; K74; K76.0 | 1 |
| **Diabetes type 1/type 2** | E10.0, E10.1; E10.9; E11.0; E11.1; E11.9 | 1 |
| **Hemiplegia** | G81; G82 | 2 |
| **Moderate to severe renal disease** | I12; I13; N00–N05; N07; N11; N14; N17–N19; Q61 | 2 |
| **Diabetes with end-organ damage type 1/type 2** | E10.2-E10.8; E11.2-E11.8 | 2 |
| **Any tumor** | C00-C75 | 2 |
| **Leukemia** | C91-C95 | 2 |
| **Lymphoma** | C81-C85; C88; C90; C96 | 2 |
| **Moderate/severe liver disease** | B15.0; B16.0; B16.2; B19.0; K70.4; K72; K76.6; I85 | 3 |
| **Metastatic solid tumor** | C76-C80 | 6 |
| **AIDS** | B21-B24 | 6 |
