# Supplementary material for: Body Mass Index of 92,027 patients acutely admitted to general hospitals in Denmark: Associated clinical characteristics and 30-day mortality
Source: PLoS One. 2018 Apr 16;13(4):e0195853. doi: 10.1371/journal.pone.0195853 (PMC5901987; doi:10.1371/journal.pone.0195853)
Supplement: S4 Table — (DOCX) [file pone.0195853.s004.docx]

**S4 Table. Prevalence of comorbidities in the Charlson Comorbidity Score, by BMI category.**

|  | **BMI<18.5 kg/m^2^** | | **BMI 18.5 to 25 kg/m^2^** | | **BMI 25 to 30 kg/m^2^** | | **BMI 30 to 35 kg/m^2^** | | **BMI 35 to 40 kg/m^2^** | | **BMI > 40 kg/m^2^** | | **Total** | |
| --- | --- | --- | --- | --- | --- | --- | --- | --- | --- | --- | --- | --- | --- | --- |
|  | N | % | N | % | N | % | N | % | N | % | N | % | N | % |
| **Overall** | 3701 | 100 | 38446 | 100 | 31093 | 100 | 12810 | 100 | 4048 | 100 | 1929 | 100 | 92027 | 100 |
| **Myocardial infarction** | 82 | 2.2 | 1327 | 3.5 | 1551 | 5 | 601 | 4.7 | 164 | 4.1 | 62 | 3.2 | 3787 | 4.1 |
| **Congestive heart failure** | 74 | 2 | 692 | 1.8 | 666 | 2.1 | 334 | 2.6 | 126 | 3.1 | 69 | 3.6 | 1961 | 2.1 |
| **Peripheral vascular disease** | 174 | 4.7 | 1233 | 3.2 | 954 | 3.1 | 359 | 2.8 | 79 | 2 | 35 | 1.8 | 2834 | 3.1 |
| **Cerebrovascular disease** | 248 | 6.7 | 3184 | 8.3 | 2970 | 9.6 | 1082 | 8.4 | 298 | 7.4 | 104 | 5.4 | 7886 | 8.6 |
| **Dementia** | 34 | 0.9 | 244 | 0.6 | 119 | 0.4 | 37 | 0.3 | 9 | 0.2 | 2 | 0.1 | 445 | 0.5 |
| **Chronic pulmonary disease** | 423 | 11.4 | 1745 | 4.5 | 1149 | 3.7 | 594 | 4.6 | 188 | 4.6 | 114 | 5.9 | 4213 | 4.6 |
| **Connective tissue disease** | 113 | 3.1 | 872 | 2.3 | 658 | 2.1 | 243 | 1.9 | 80 | 2 | 39 | 2 | 2005 | 2.2 |
| **Ulcer disease** | 95 | 2.6 | 579 | 1.5 | 412 | 1.3 | 176 | 1.4 | 53 | 1.3 | 21 | 1.1 | 1336 | 1.5 |
| **Mild liver disease** | 34 | 0.9 | 261 | 0.7 | 221 | 0.7 | 118 | 0.9 | 24 | 0.6 | 16 | 0.8 | 674 | 0.7 |
| **Diabetes I and II** | 51 | 1.4 | 700 | 1.8 | 731 | 2.4 | 544 | 4.2 | 243 | 6 | 159 | 8.2 | 2428 | 2.6 |
| **Hemiplegia** | 17 | 0.5 | 78 | 0.2 | 52 | 0.2 | 14 | 0.1 | 8 | 0.2 | 4 | 0.2 | 173 | 0.2 |
| **Moderate to severe renal disease** | 70 | 1.9 | 637 | 1.7 | 584 | 1.9 | 275 | 2.1 | 95 | 2.3 | 50 | 2.6 | 1711 | 1.9 |
| **Diabetes with end organ damage** | 28 | 0.8 | 360 | 0.9 | 391 | 1.3 | 332 | 2.6 | 146 | 3.6 | 101 | 5.2 | 1358 | 1.5 |
| **Any tumor** | 430 | 11.6 | 3406 | 8.9 | 2413 | 7.8 | 881 | 6.9 | 215 | 5.3 | 102 | 5.3 | 7447 | 8.1 |
| **Leukemia** | 12 | 0.3 | 131 | 0.3 | 98 | 0.3 | 26 | 0.2 | 14 | 0.3 | 2 | 0.1 | 283 | 0.3 |
| **Lymphoma** | 24 | 0.6 | 247 | 0.6 | 166 | 0.5 | 41 | 0.3 | 13 | 0.3 | 7 | 0.4 | 498 | 0.5 |
| **Moderate to severe liver disease** | 9 | 0.2 | 95 | 0.2 | 87 | 0.3 | 33 | 0.3 | 6 | 0.1 | 3 | 0.2 | 233 | 0.3 |
| **Metastatic solid tumor** | 149 | 4 | 1056 | 2.7 | 652 | 2.1 | 216 | 1.7 | 49 | 1.2 | 21 | 1.1 | 2143 | 2.3 |
| **AIDS** | 8 | 0.2 | 41 | 0.1 | 14 | 0 | 8 | 0.1 | 3 | 0.1 | 0 | 0 | 74 | 0.1 |
