# Supplementary material for: Body Mass Index of 92,027 patients acutely admitted to general hospitals in Denmark: Associated clinical characteristics and 30-day mortality
Source: PLoS One. 2018 Apr 16;13(4):e0195853. doi: 10.1371/journal.pone.0195853 (PMC5901987; doi:10.1371/journal.pone.0195853)
Supplement: S5 Table — (DOCX) [file pone.0195853.s005.docx]

**S5 Table. Anatomical Therapeutic Chemical Classification System codes (ATC-codes) for prescription medications from the Danish National Prescription Health Service Database.**

**ATC codes for prescriptions: Medications taken continuously**

| **Type of medications** | **ATC-codes** |
| --- | --- |
| **Medications for gastric acid-related disorders** | A02A-A02B |
| **Glucose-lowering medications:** |  |
| Insulin analogues: | A10A |
| Metformin (Biguanides): | A10BA, A10BD02, A10BD03, A10BD05, A10BD07, A10BD08, A10BD10, A10BD11, A10BD13 |
| Other glucose-lowering medications: |  |
| Sulfonylureas | A10BB, A10BD04, A10BD02, A10BD06, A10BD01 |
| Meglitinides | A10BX02 |
| α-glucosidase inhibitor | A10BF |
| Glitazone | A10BG, A10BD03, A10BD04, A10BD05, A10BD06, A10BD09, A10BD12 |
| Inhibitors of dipeptidyl peptidase 4 (from 2007) | A10BH, A10BD07, A10BD08, A10BD09, A10BD10, A10BD11, A10BD12, A10BD13 |
| Glucagon-like peptide-1 analogue (from 2007) | A10BX04, A10BX07, A10BX10 |
| Carbamoylmethyl benzoic acid derivate | A10BX02 |
| Sodium/glucose cotransporter 2 inhibitors | A10BX10, A10BX11, A10BX12 |
| **Lipid-modifying medications:** | C10 |
| Statins | C10AA, C10 BA, C10BX |
| Fibrates | C10AB |
| Anion exchangers | C10AC |
| Nicotinic acid | C10AD |
| **Antihypertensive medications:** | C02A- C02C, C03A-E, C07-C09X |
| **Antidepressants:** |  |
| Lamotrigine: | N03AX09 |
| Tricyclic antidepressants: | N06AA |
| Selective serotonin reuptake inhibitors: | N06AB |
| Monoamine oxidase inhibitors: | N06AF |
| Nordarenergic and Specific Serotonergic Antidepressants: | N06AX |

**ATC codes for prescriptions: Medications taken episodically**

| **Type of medications** | **ATC codes** |
| --- | --- |
| **Glucocorticoids:** |  |
| Systemic: | H02AB, |
| Inhalation: | R03BA |
| Intestinal: | A07EA |
| **Antibiotics:** | A07AA, J01AA02- J05AB11 |
| **Prescription painkillers:** |  |
| NSAIDs | M01 - M02 |
| Opioids | N02A |
| Over the counter | N02B |
| Migraine | N02C |
| **Inhalants for obstructive airway diseases:** | R03 |
