# Supplementary material for: Body Mass Index of 92,027 patients acutely admitted to general hospitals in Denmark: Associated clinical characteristics and 30-day mortality
Source: PLoS One. 2018 Apr 16;13(4):e0195853. doi: 10.1371/journal.pone.0195853 (PMC5901987; doi:10.1371/journal.pone.0195853)
Supplement: S6 Table — (DOCX) [file pone.0195853.s006.docx]

**S6 Table. Prevalences of prehospital medication users in the cohort according to BMI category.**

|  | **BMI<18.5 kg/m^2^** | **BMI 18.5 to 25 kg/m^2^** | **BMI 25 to 30 kg/m^2^** | **BMI 30 to 35 kg/m^2^** | **BMI 35 to 40 kg/m^2^** | **BMI > 40 kg/m^2^** | **Total** |
| --- | --- | --- | --- | --- | --- | --- | --- |
| **Overall:** | 3701 (100) | 38,446 (100) | 31093 (100) | 12810 (100) | 4048 (100) | 1929 (100) | 92,027 (100) |
| **Any medication** | 2836 (76.6) | 26,494 (68.9) | 2,2682 (72.9) | 10,003 (78.1) | 3312 (81.8) | 1612 (83.6) | 66,939 (72.7) |
| **Antihypertensive medications** | 489 (13.2) | 5286 (13.7) | 5690 (18.3) | 2796 (21.8) | 957 (23.6) | 415 (21.5) | 15,633 (17) |
| **Glucose-lowering medications** | 144 (3.9) | 2112 (5.5) | 2817 (9.1) | 1907 (14.9) | 815 (20.1) | 456 (23.6) | 8251 (9) |
| **Lipid-lowering medications** | 546 (14.8) | 7263 (18.9) | 8294 (26.7) | 3962 (30.9) | 1292 (31.9) | 563 (29.2) | 21,920 (23.8) |
| **Antidepressant and anxiolytic medications** | 852 (23) | 6158 (16) | 4973 (16) | 2411 (18.8) | 890 (22) | 469 (24.3) | 15,753 (17.1) |
| **Prescription painkillers** | 1406 (38) | 11,999 (31.2) | 10,286 (33.1) | 5078 (39.6) | 1766 (43.6) | 893 (46.3) | 31,428 (34.2) |
| **Inhalants for obstructive airway diseases** | 743 (20.1) | 4279 (11.1) | 3253 (10.5) | 1616 (12.6) | 592 (14.6) | 315 (16.3) | 10,798(11.7) |
| **Treatment for gastric acid related disorders** | 671 (18.1) | 5693 (14.8) | 5430 (17.5) | 2544 (19.9) | 842 (20.8) | 412 (21.4) | 15,592 (16.9) |
| **Antibiotic medications** | 1411 (38.1) | 11,716 (30.5) | 8913 (28.7) | 3991 (31.2) | 1393 (34.4) | 754 (39.1) | 28,178 (30.6) |
| **Glucocorticoid medications** | 434 (11.7) | 2926 (7.6) | 2212 (7.1) | 1010 (7.9) | 318 (7.9) | 160 (8.3) | 7060 (7.7) |
